# Olfactory-Guided Behavior Uncovers Imaging and Molecular Signatures of Alzheimer’s Disease Risk

**DOI:** 10.3390/brainsci15080863

**Published:** 2025-08-13

**Authors:** Hae Sol Moon, Zay Yar Han, Robert J. Anderson, Ali Mahzarnia, Jacques A. Stout, Andrei R. Niculescu, Jessica T. Tremblay, Alexandra Badea

**Affiliations:** 1 Quantitative Imaging and Analysis Laboratories, Radiology Department, Duke University Medical Center, Durham, NC 27710, USA; haesol.moon@duke.edu (H.S.M.); zayyar.han@duke.edu (Z.Y.H.); robert.j.anderson@duke.edu (R.J.A.); amahzarn@stanford.edu (A.M.); jacques.stout@duke.edu (J.A.S.); jtrembl4@tennessee.edu (J.T.T.); 2 Biomedical Engineering Department, Pratt School of Engineering, Duke University, Durham, NC 27710, USA; 3 Brain Imaging and Analysis Center, Duke University Medical Center, Durham, NC 27710, USA; 4 Pediatric Department, Stanford University Medical Center, Palo Alto, CA 94305, USA; 5 Neurology Department, Duke University Medical Center, Durham, NC 27710, USA

**Keywords:** olfactory behavior, APOE, Alzheimer’s disease, neuroimaging, connectomics, biomarkers

## Abstract

**Background/Objectives:** Olfactory impairment has been proposed as an early marker for Alzheimer’s disease (AD), yet the mechanisms linking sensory decline to genetic and environmental risk factors remain unclear. We aimed to identify early biomarkers and brain network alterations associated with AD risk by multimodal analyses in humanized APOE mice. **Methods:** We evaluated olfactory behavior, diffusion MRI connectomics, and brain and blood transcriptomics in mice stratified by APOE2, APOE3, and APOE4 genotypes, age, sex, high-fat diet, and immune background (HN). Behavioral assays assessed odor salience, novelty detection, and memory. Elastic Net-regularized multi-set canonical correlation analysis (MCCA) was used to link behavior to brain connectivity. Blood transcriptomics and gene ontology analyses identified peripheral molecular correlates. **Results:** APOE4 mice exhibited accelerated deficits in odor-guided behavior and memory, especially under high-fat diet, while APOE2 mice were more resilient (ANOVA: APOE x HN, F(2, 1669) = 77.25, *p* < 0.001, eta squared = 0.08). Age and diet compounded behavioral impairments (diet x age: F(1, 1669) = 16.04, *p* < 0.001). Long-term memory was particularly reduced in APOE4 mice (APOE x HN, F(2,395) = 5.6, *p* = 0.004). MCCA identified subnetworks explaining up to 24% of behavioral variance (sum of canonical correlations: 1.27, 95% CI [1.18, 1.85], *p* < 0.0001), with key connections involving the ventral orbital and somatosensory cortices. Blood eigengene modules correlated with imaging changes (e.g., subiculum diffusivity: r = −0.5, *p* < 1 × 10^−30^), and enriched synaptic pathways were identified across brain and blood. **Conclusions**: Olfactory behavior, shaped by genetic and environmental factors, may serve as a sensitive, translatable biomarker of AD risk. Integrative systems-level approaches reveal brain and blood signatures of early sensory–cognitive vulnerability, supporting new avenues for early detection and intervention in AD.

## 1. Introduction

Alzheimer’s disease (AD) is a complex and multifactorial neurodegenerative disorder that primarily affects the aging population and remains irreversible, with no definitive cure to date [1]. It is marked by a progressive deterioration of cognitive functions, including memory deficits and behavioral changes. Hallmark features of AD pathology include the aggregation of amyloid-β plaques, hyperphosphorylated tau tangles, brain atrophy, and the disruption of neural circuits [2,3,4]. The disease has a prolonged preclinical phase during which subtle physiological and behavioral abnormalities emerge, about 10 to 15 years before obvious symptoms and clinical diagnosis [5]. This extended prodromal window underscores a critical need for early biomarkers predictive of cognitive decline, and for identifying individuals at risk. Determining the initial circuit-level perturbations that precede irreversible neurodegeneration [5,6] may offer new targets for more effective interventions.

One promising target is the olfactory circuit, as olfactory dysfunction has been proposed as a low-cost and accessible biomarker for early-stage detection [7,8]. Individuals at risk for or in the early stages of AD often demonstrate impairments in odor detection, discrimination, identification, and memory—well before the onset of clinically diagnosable cognitive decline [9,10]. This early vulnerability is partly attributable to the unique anatomy of the olfactory system, which is directly and densely connected to key brain regions implicated in AD pathology—including the entorhinal cortex, amygdala, and hippocampus [11]. These regions are among the first to undergo neurodegenerative changes and pathological protein accumulation in both human patients and animal models of AD [11,12]. In clinical research, olfactory function is commonly assessed through standardized cognitive tests such as the University of Pennsylvania Smell Identification Test (UPSIT), Sniffin’ Sticks, and AROMHA Brain Health Test (ABHT) [8,13,14]. These tasks not only measure perceptual sensitivity but also engage higher-order memory systems, informing on early sensory–cognitive decline [15]. Given the functional and anatomical relationship between olfaction and memory, olfactory testing provides a uniquely lens through which to investigate the earliest neural disruptions associated with AD [16]. However, while olfactory behavior is known to involve networks of brain regions, the relationship between behavior performance on these odor-based tasks and brain connectivity remains poorly understood in the context of AD. A comprehensive framework that integrates olfactory tasks with systems-level neuroimaging, such as diffusion MRI based connectomics, could uncover new insights into the early sensory–cognitive changes in AD and their neural circuits that underlie them.

The clinical relevance of olfactory dysfunction in AD has spurred the refinement of mouse behavioral assays, yet key methodological hurdles remain. For example odor memory is long-lasting but can easily be masked by dominant visual cues or by odors that are insufficiently novel [17]. Still, animal models can help advance our understanding of AD, dissecting the complex interplay between genetic and environmental risk factors and their impact on behavior and associated brain circuits. Mouse models provide a well-controlled experimental platform that allows for precise manipulation of variables that are difficult to isolate in human populations—like diet [18,19,20,21] or genetic background. This level of experimental control enables causal inferences and the ability to investigate mechanistic underpinnings of disease-related phenotypes. Mice expressing the human APOE4 allele model the highest known genetic risk for AD, relative to the control APOE3 allele and the protective APOE2 allele [22]. APOE4 mice show olfactory dysfunction and olfactory memory impairments compared to APOE2 and APOE3 mice [23], yet the mechanisms explaining the relationships between APOE, olfaction and AD risk are not fully understood. High-resolution imaging offers the possibility to reveal early biomarkers based on alterations in the circuitry of olfactory regions, which are connected to areas involved in motivated behavior and cognition [24], at stages that precede overt neurodegeneration [25,26] and pathological load. This motivates our study to uncover the mechanisms by which brain connectivity and microstructural alterations associated with AD risk influence olfactory behavioral performance—particularly long-term memory [27]—and to determine whether these mechanisms can be detected through peripheral molecular markers.

We aim to investigate how AD-related genetic and modifiable risk factors impact olfactory behaviors in mice, and how these behavioral phenotypes relate to alterations in brain connectivity. Specifically, we test odor preference, reaction to novel odors, and memory in mice that model various combinations of risk factors. These mice carry the humanized apolipoprotein E (APOE) gene (ε2, ε3, and ε4) to model different levels of AD genetic risk; and either mouse Nos2 or humanized nitric oxide synthase 2 (hNOS2) to mimic human-like immune responses; sex (male and females); age (middle-a and old aged); and environmental stressors such as high-fat diet [28,29,30]. By studying these factors both independently and in combination, we aim to identify not only which risk variables impair olfactory performance, but also which synergistic combinations lead to more pronounced olfactory behavioral decline. To integrate these multimodal data sources, we employed a multistep approach: (1) MCCA linked imaging networks to risk factors and behavior; (2) we then connected those networks to blood transcriptomic modules to explain microstructural variance in olfactory–memory circuits; (3) we integrated these findings to create a mechanistic bridge from behavior to brain networks to peripheral signatures. We paired behavioral assessments with high-resolution ex vivo diffusion MRI-based connectomics to identify brain regions and networks [31] for which structural integrity and connectivity correlate with olfactory behavior. We hypothesized our hits include memory and sensory integration circuits. Our goals were: (1) to determine how genetic and environmental risk factors for AD impact odor-related behavioral markers; (2) to uncover the brain circuits that underlie these behavioral changes, offering insights into the early pathophysiology of AD; (3) to investigate mechanistic changes in peripheral markers that may offer a noninvasive insight into central processes. Finally, we assessed whether regional differences in brain gene expression can be mapped using peripheral blood transcriptomics. Our findings showed that we can detect such central marker changes using peripheral markers, and this method offers rich insight into pathways altered in individuals with different risk profiles for AD. We hope our study will contribute to the development of translatable early biomarkers and add methods to improve our mechanistic understanding of how diverse risk factors interact to shape disease trajectories.

## 2. Materials and Methods

### 2.1. Animals

Animal procedures were approved by the Duke Institutional Animal Care and Use Committee. To model genetic risk for late-onset Alzheimer’s disease (LOAD), we used animals lacking the endogenous mouse APOE gene and homozygous for one of the three major human APOE alleles: the high-risk APOE4, the neutral APOE3, and the protective APOE2. These lines were crossed with mice where the mouse Nos2 gene was replaced by the human NOS2 gene (HuNOS2tg/mNos2−/−, termed HN). This replacement models the reduced NOS2 expression seen in human macrophages compared to murine counterparts, aligning redox homeostasis more closely with the human. Both sexes were included. Mice were naturally aged, with dietary exposures to either regular chow (2001 Lab Diet) or a high-fat diet (HFD; D12451i, Research Diets, New Brunswick, NJ, USA) for the last 4 months of life. Control diet consisted of 13.6 kcal% fat, 57.5 kcal% carbohydrate (3.25 kcal% sucrose), and 28.9 kcal% protein, while HFD contained 45 kcal% fat (39 kcal% from lard; 5 kcal% from oil), 35 kcal% carbohydrate (17 kcal% sucrose), and 20 kcal% protein. Mice had ad libitum access to food and water.

**Figure 1:**
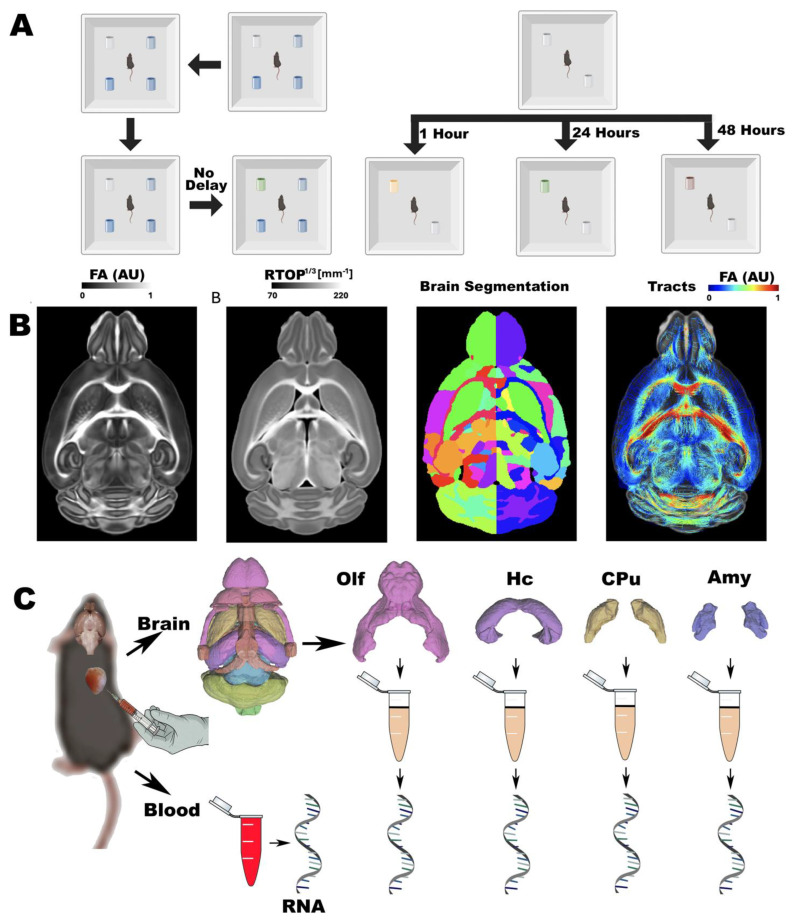
Experiments included olfactory guided behavior, MRI, transcriptomics of blood and brain regions. (**A**) Olfactory guided behavior tests: Odor preference during habituation: Mice freely explored an open field containing four objects, each infused with different concentrations of lemon odorant. Habituation–Dishabituation task: Mice were exposed to four consecutive trials with the same odor (lemon), followed by a dishabituation trial in which the lemon-scented object was replaced with one scented with vanilla. Memory tests: To assess short-term, long-term, and extended-term olfactory memory, mice were first exposed to two objects containing the same novel odor. At 1 h, 24 h, and 48 h after initial exposure, one of the odors was replaced with a new one to test recognition. (**B**) High resolution DWI images were used to reconstruct parametric maps and tractography based connectomes. FA map, a traditional DTI model metric. Return-to-origin probability map (RTOP), an advanced MAP model metric. Brain segmentation of 332 regions. Tractography was used in conjunction with region labels to reconstruct connectomes. (**C**) Transcriptomic experiments were conducted using peripherally collected blood and on several brain regions, including the olfactory bulbs/piriform cortex (Olf), the hippocampus (Hc), caudate putamen (CPu), and amygdala (Amy).

The animal cohorts used for behavioral testing are described in Table 1. A total of 465 mice were included in the behavioral analysis, stratified by APOE genotype (APOE2, APOE3, APOE4), diet (control or high-fat diet), sex, age group (younger: ~12 months, older: ~18 months), and NOS2 genetic background (mouse NOS2 [mNOS2] or human NOS2 transgene [HN]). A subset of 175 mice was used for imaging, as described in Table S1. An independent set of 139 mice were used for blood RNA-seq, as described in Table S2. From these last cohort, a subset was used for brain RNA-seq. We collected blood from a cohort partly overlapping with the MRI cohort (N = 130), while tissue samples were collected from up to 34 animals per brain region, specifically: hypothalamus (*n* = 34), cingulate cortex (*n* = 32), frontal cortex (*n* = 32), hippocampus (*n* = 27), amygdala (*n* = 8), brainstem (*n* = 7), caudate putamen (*n* = 8), thalamus (*n* = 8), olfactory bulb (*n* = 7), substantia nigra (*n* = 6), colliculus (*n* = 6), and cerebellum (*n* = 3), which were compared with blood samples from the same animals, to examine patterns of shared variation. The cohort was balanced across APOE genotype, sex, diet, age range, and NOS2 status. The experiments are shown in Figure 1.

### 2.2. Behavior Assessments

#### 2.2.1. Odor Preference and Habituation–Dishabituation Test

Mice were acclimated to the testing room for 30 min prior to the start of behavioral testing (Figure 1A). On Day 1, each mouse was placed in the center of a square Plexiglas open-field arena (40 × 40 cm) and allowed to explore freely for 10 min. Between subjects, the arena was wiped with 70% ethanol and water. We recorded exploration time for various zones of the apparats using ANY-maze (Stoelting Co., Wood Dale, IL, USA) [32], with experimenters blinded to group identity.

We assessed odor preference and olfactory sensitivity using a series of trials involving different dilutions of lemon scent in mineral oil, as well as mineral oil alone. The odorants were presented within custom-designed, 3D-printed hollow objects featuring perforations to allow scent dispersion. Food-grade lemon essence was diluted in mineral oil at concentrations of 0 (vehicle), 0.001, 0.01, and 0.1 *v*/*v* (volume/volume). Each test lasted 2 min, during which four odorized objects were simultaneously presented. To reduce potential location bias, the positions of the objects were pseudorandomized between sessions. Each condition was tested in four consecutive trials, followed by a dishabituation trial, where the familiar odor (lemon) was replaced with a novel odor (vanilla or almond, depending on cohort) to assess novelty detection. Preference ratios were recorded based on the time spent investigating each object.

The dishabituation index was calculated using Trial 4 and the last trial with introduction of the novel odor at Trial 5, with Trials 1–3 serving the habituation purpose. Anhedonia-like behavior was estimated as the difference between tracked time and time spent engaging with odorized objects; and as the proportion of total tracked time that mice spent not engaging with odorized objects: Anhedonia = (Tracked time—Total odor exploration time) ÷ Tracked time. Higher values reflect reduced interest in odor stimuli (anhedonia-like behavior), while lower values reflect greater hedonic engagement. Periods of immobility (freezing) were retained in the calculation because they may reflect diminished hedonic drive and contribute meaningfully to the overall measure.

#### 2.2.2. Novel Odor Memory

We analyzed odor-based memory tests using a protocol that began with two identical odors, followed by the introduction of a novel scent after specific time delays (1 h, 24 and 48 h). Mice were first exposed to two objects scented with coconut oil across three identical habituation trials. To assess short-term memory, one coconut-scented object was replaced in trial 4 with an almond-scented object after a 1 h delay. For long-term memory (24 h delay), the novel scent was changed to anise, and for extended-term memory (48 h delay), banana was used. Across all tests, the positions of the familiar and novel odor objects were alternated to reduce spatial bias and isolate olfactory-driven responses, following procedures adapted from [33,34].

The following metrics were used to quantify behavior:

$$Preference\;Ratio=\;\frac{N_{o}}{Total\;time\;at\;all\;objects}$$

$$Recognition\;Index\;\left(RI\right)=\frac{N_{o}}{N_{o}+F_{o}}$$

$$Dishabituation\;Index\;\left(DI\right)=\frac{N_{o}-F_{o}}{N_{o}+F_{o}}$$

where $N_{o}$ is the investigation time at the novel odor, and $F_{o}$ is the time at the familiar odor. Investigation time is the duration (s) for which the nose centroid of the mouse remained within a 2 cm radius of the object to measure close investigation of the odorant object.

### 2.3. Image Acquisition and Processing

After conclusion of the behavior tests, animals were sacrificed to prepare specimens for ex vivo imaging. Animals were euthanized under a surgical plane of anesthesia (100 mg/kg ketamine and 10 mg/kg xylazine) and perfused transcardially via the left ventricle with 0.9% saline (~8 mL/min for 5 min), followed by 10% neutral buffered formalin phosphate containing 10% Gadoteridol (ProHance, Bracco Diagnostics, Milan, Italy) at the same flow rate and duration. Brains were left within the skull to preserve anatomical integrity and stored in formalin for 12 h, followed by rehydration in 0.5% Gadoteridol in phosphate-buffered saline at 4 °C. Heads were trimmed of extraneous tissue and placed in MRI-compatible tubes filled with perfluoropolyether (Galden Pro) for susceptibility matching.

MRI acquisitions included high-resolution diffusion-weighted protocols and were acquired using on a 9.4T high-field scanner (Figure 1B) using a 3D spin-echo sequence (TR/TE = 100 ms/14.2 ms, matrix = 420 × 256 × 256, FOV = 18.9 × 11.5 × 11.5 mm^3^, resolution = 45 μm isotropic), as in [28]. Acquisition was accelerated with 8-fold compressed sensing. The protocol included 46 diffusion directions across two shells (b = 2000 and 4000 s/mm^2^) and five b0 images.

Diffusion tensor metrics including fractional anisotropy (FA), axial/radial/mean diffusivity orientation distribution functions, and tractograms were reconstructed using MRtrix3 [35], and filtered via SIFT. The MAP (mean apparent propagator) indices including mean squared displacement, non-Gaussianity, q-space inverse variance, and return-to-origin probability (RTOP) were estimated using DiPy [36]. Regional volumes were calculated as percent of intracranial volume, following brain segmentation and parcellating each brain into 332 anatomically defined regions, using SAMBA [37], and a publicly shared mouse brain atlas [28,38]. https://zenodo.org/record/8377684 (accessed on 31 July 2025). Structural connectomes were constructed as adjacency matrices representing streamline counts between region pairs.

### 2.4. Transcriptomics

RNA was extracted from blood and select brain regions using RNeasy Mini Kit (QIAGEN, Hilden, Germany) (Figure 1C). The brain regions included the Piriform Cortex, Amygdala, Hippocampus, Hypothalamus, Caudate Putamen, Cingulate Cortex, Frontal Association Cortex, Thalamus, Brainstem, Substantia Nigra, Superior and Inferior Colliculi, and Cerebellum. RNA-Seq experiments were conducted at the Duke Sequencing and Genomic Technologies Shared Resource Core to identify genes differentially expressed with age and sex. The RNA quality was assessed with a NanoDrop 2000 spectrophotometer. Samples were processed on an Illumina NovaSeq600 S2 platform, with 50 bp PE full flow cell, utilizing NuGEN mRNA-Seq with Any Deplete Globin. Sequencing data underwent quality control through FastQC, adhering to a Phred score cutoff above 20. As no adapter sequences were found adjacent to transcript reads, data trimming was not required. We aligned raw RNA-seq reads to a Mus musculus reference transcriptome, and the Salmon program [39] was utilized to quantify transcript abundance, as in [40]. Transcriptomes were normalized before deriving 10 PCs, termed the eigengene, as summaries of expression measures. We performed gene ontology (GO) enrichment on the top-loading genes for each principal component (PC). For each PC, genes were ranked by the absolute value of their loading and the top 200 retained. Mouse gene symbols were mapped to Entrez IDs via org.Mm.eg.db, and any PC with fewer than 10 mapped IDs was omitted. To identify biological processes associated with PC modules, we performed GO analysis using the clusterProfiler package version 4.12.6 in R 4.4.1, querying the GO Biological Process (GO:BP) category. Multiple testing correction was applied using the Benjamini–Hochberg False Discovery Rate (FDR) method. Pathways with an FDR-adjusted *p*-value < 0.05 were considered statistically significant and included in further analyses or visualization.

### 2.5. Multivariate Modeling

To examine shared variance between brain structural connectivity and multidomain traits, we implemented a two-stage multivariate pipeline combining Elastic Net regression and Multi-set Canonical Correlation Analysis (MCCA), expanding upon previous work [40].

Dimensionality reduction was first performed using Elastic Net regularization [41]. The connectome matrix *X* (nodes × edges) was regressed onto behavioral outcomes *Y* using a penalty that blends *L1* and *L2* norms:

$$\mathrm{loss}=\frac{1}{2n}|Y-X\beta {|}_{2}^{2}+\lambda \left(\alpha |\beta {|}_{1}+\frac{1-\alpha }{2}|\beta {|}_{2}^{2}\right)$$

Here, *β* is the vector of coefficients to be learned, *λ* is the regularization parameter, and *α* (set to 0.5) balances sparsity (*L1* norm) and ridge penalty (*L2* norm). The optimal *λ* was identified via 10-fold cross-validation, retaining only connectivity features with non-zero coefficients, to ensure ensured robust dimensionality reduction prior to multivariate modeling.

We applied MCCA to identify latent dimensions that link three domains: (1) structural connectomes, (2) olfactory-guided behavioral responses (odor salience, anhedonia, odor memory), and (3) AD risk traits (APOE genotype, diet, immune background, age, and sex). MCCA finds maximally correlated linear projections across datasets by solving:

$$max_{w_{1},w_{2},\ldots ,w_{m}}\sum_{i<j}corr\left(X_{i}w_{i},X_{j}w_{j}\right)$$

where *X(k)* is the standardized data matrix for domain *k*, and *w(k)* is its canonical vector. Each matrix was scaled and entered separately to preserve within-domain structure while allowing cross-domain alignment. The resulting canonical scores were back-projected into brain space by expanding feature indices to the original connectome matrix, enabling the identification of brain subnetworks that contribute most strongly to cross-domain variance. This integrative approach allowed us to extract distributed network-level signatures of AD risk and behavior, reflecting early disruption of brain–olfactory-guided behavior alignment across systems.

### 2.6. Peripheral Molecular Mechanisms Associated with Brain Imaging Features

To gain insight into molecular mechanisms underlying image feature changes we integrated complementary data streams in the same cohort of animals: (1) regional diffusion-MRI and volumetric metrics extracted from 3D structural images, and (2) blood-derived gene-expression modules. For focused analysis of olfactory-memory circuits, we selected eight a priori brain regions: the Piriform Cortex, Amygdalo-piriform Transition Area, Posterolateral Cortical Amygdaloid Area, Perirhinal Cortex, Hippocampus, Postsubiculum, Parasubiculum, and Ventral Orbital Cortex.

To assess gene–imaging coupling, we merged imaging and transcriptomic data with one row per subject–region–metric–eigengene combination. Within each brain region, both imaging metrics and eigengenes were z-scored, allowing regression slopes to be interpreted as Pearson correlation coefficients. Linear models were fit for each of the 560 metric–region–eigengene combinations (8 regions × 10 eigengenes × 7 imaging metrics), modeling eigengene expression as a function of imaging metrics. *p*-values were corrected using the Benjamini–Hochberg procedure, and associations with FDR <0.05 were retained for downstream analysis.

We selected the top five regions per metric–eigengene pair with the strongest correlations and visualized signed effect sizes (*β*), –log_10_(FDR) values, and absolute correlations across a grid of metric × eigengene facets. For functional interpretation, we performed GO Biological Process enrichment using the top 100 genes from each module with the gprofiler2 package without initial *p*-value filtering to capture strong and moderate associations. Results were visualized using ggplot2 3.5.1, displaying the top 10 enriched pathways per module based on –log_10_(*p*-value).

To identify systemic molecular signatures associated with regional brain vulnerability in AD, we analyzed the correlation structure between gene expression PCs from blood and brain regions (hypothalamus, hippocampus, frontal cortex, and cingulate cortex). For each region, the top PCs from the brain dataset were correlated against the top PCs from the blood dataset using both Pearson and Spearman correlation tests. All *p*-values from these pairwise tests were adjusted for multiple comparisons using the Benjamini–Hochberg procedure. For each significant brain–blood PC pair, we selected the top 200 genes with the highest absolute loading values in each PC. The intersection of these two gene sets (brain and blood) was used for Gene Ontology (GO, Biological Process) enrichment analysis, performed using the clusterProfiler package (mouse: org.Mm.eg.db; human: org.Hs.eg.db; keyType = “SYMBOL”; BH adjustment; *p* < 0.05). Only intersections comprising at least five genes were considered for enrichment testing. Enriched GO terms and their constituent genes were recorded for downstream interpretation. All computational steps, including correlations, FDR correction, and GO enrichment, were parallelized to expedite processing across regions.

This intersection-based approach allows for the identification of molecular pathways that are systemically reflected across both blood and brain tissues, capturing not only neural but also metabolic, apoptotic, and immune-related biological processes. By integrating two tissue types, the method naturally detects a broader spectrum of systemic pathways, offering mechanistic insight into processes underlying shared variance between brain regions and peripheral blood. The gene sets and pathways identified from the brain–blood PC intersections were then used as input for subsequent region-to-region analyses within the brain. We further prioritized pathways relevant to the olfactory region, extracting and interpreting those biological processes that showed shared variation detectable in both brain and blood.

### 2.7. Statistical Analysis

All analyses were performed in R 4.4. Data wrangling and visualization used the tidyverse (ggplot2 3.5.1, ggpubr 0.6.0), and computations were parallelized with future 1.34.0 and furrr 0.3.1 to accelerate ROI-level models.

For behavioral outcomes (head-time, preference ratio, normalized odor exploration, AUC_raw, AUC, anhedonia metrics, dishabituation index), we used linear mixed-effects models (lmer, lme4) when data included repeated measures (odor concentration or trial number as the within-subject term; APOE genotype, diet, age category, HN status, and sex as fixed factors; mouse ID as a random effect: outcome ~ Odor_Concentration + APOE * Diet * Age * HN * Sex + (1|Mouse_ID)). Single-measure endpoints (e.g., dishabituation index, single timepoint memory scores) were analyzed with linear models (lm) using the same fixed-effect structure (outcome ~ APOE * Diet * Age * HN * Sex). Type III ANOVA tables provided omnibus significance. Tukey-adjusted post hoc tests (emmeans) were used for all-pairwise contrasts of categorical factors with more than two levels, while Sidak-adjusted tests were reserved for planned comparisons (e.g., APOE3 vs. APOE4). Partial η^2^ and Cohen’s f (with 95% confidence intervals) were extracted for all fixed terms.

For brain regional statistics (diffusion metrics: FA, MD, RD, MAP-MRI indices, and regional volumes), we ran general linear models for each ROI (outcome ~ APOE * Diet * Age * HN * Sex), computed F-tests via the ANOVA function, and extracted Cohen’s f and partial η^2^. Multiple comparisons across ROIs and imaging metrics were controlled with Benjamini–Hochberg false discovery rate (FDR) correction at 5%.

For multivariate modeling, we used Elastic Net regression for dimensionality reduction, followed by multi-set canonical correlation analysis (MCCA) to link behavior, imaging, and AD risk traits. The optimal Elastic Net penalty (*λ*) was identified via 10-fold cross-validation, and retained features entered the MCCA model. 1000 bootstrap iterations estimated confidence intervals for summed canonical correlations.

For gene-expression analyses, eigengene modules were derived via PCA of normalized RNA-seq data. We used linear models to test associations between eigengenes and regional imaging metrics (one model per metric–region–eigengene combination), applying FDR correction across all tests. Gene ontology enrichment (clusterProfiler, gprofiler2) was performed on the top-loading genes from each eigengene module, with Benjamini–Hochberg adjustment (FDR < 0.05).

Across all analyses, *α* = 0.05 defined statistical significance after correction where applicable. Results were visualized as forest plots, chord diagrams, and clustered heatmaps of Cohen’s f and other effect sizes.

## 3. Results

### 3.1. Olfactory Guided Behavior

To evaluate the influence of AD risk factors on olfactory-guided behavior, we assessed odor preference, anhedonia-like responses, and exploratory dynamics in mice stratified by APOE genotype, age, sex, diet, and immune background (hNOS2 vs. mNos2). Across behavioral assays, we observed consistent and converging evidence that both intrinsic (e.g., genotype, sex) and extrinsic (e.g., diet, age, immune status) factors interactively modulate olfactory behavior.

#### 3.1.1. Odor Salience

**Figure 2:**
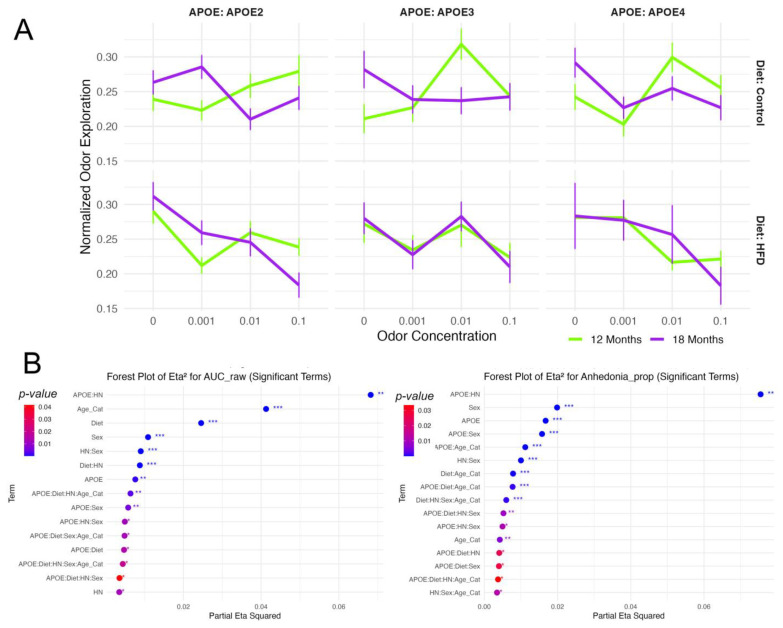
Odor salience is modulated by genotype, age, and diet. (**A**) Normalized odor exploration across four odor concentrations stratified by APOE genotype, age (12 vs. 18 months), and diet (control vs. high-fat). Each subplot represents group means ± SE. (**B**) Forest plots of partial eta squared (η^2^) effect sizes for significant terms (*p* < 0.05) in models assessing raw odor salience (AUC) and proportional Anhedonia. Terms are sorted by η^2^, with significance stars indicating thresholds: * *p* <  0.05, ** p < 0.01, *** p < 0.001.

Odor salience (Figure 2), measured via normalized odor exploration across escalating concentrations of lemon odorant, showed a robust main effect of concentration (F(3, 1669) = 7.46, *p* < 0.001, η^2^ = 0.0116, f = 0.108), indicating stimulus-dependent modulation of exploratory behavior. A significant concentration × age interaction (F(3, 1669) = 7.77, *p* < 0.001, η^2^ = 0.0121) suggested age-related changes in odor tuning. Additional interactions with biological traits were significant, including concentration × diet (F(3, 1669) = 6.67, *p* < 0.001, η^2^ = 0.0104), concentration × APOE × diet (F(6, 1669) = 3.26, *p* = 0.003, η^2^ = 0.0102), and concentration × APOE × HN (F(6, 1669) = 2.22, *p* = 0.039, η^2^ = 0.0069). A five-way interaction (concentration × APOE × age × HN × sex) also reached significance (F(6, 1669) = 3.86, *p* < 0.001, η^2^ = 0.0120), highlighting a highly multifactorial regulation of odor-guided responses.

#### 3.1.2. Anhedonia

Anhedonia-like behavior was significantly modulated by APOE × HN interactions. For the absolute time not spent with odorized objects, the interaction was highly significant (F(2, 1669) = 77.25, *p* < 0.001, η^2^_partial = 0.079, f = 0.292), with consistent effects in the proportional analysis (F(2, 1669) = 73.77, *p* < 0.001, η^2^_partial = 0.075). Main effects of APOE (F(2, 1669) = 41.46, *p* < 0.001, η^2^_partial = 0.044), sex (F(1, 1669) = 32.69, *p* < 0.001, η^2^_partial = 0.018), and diet × age (F(1, 1669) = 21.66, *p* < 0.001, η^2^_partial = 0.012) were also observed. Several higher-order interactions contributed to the behavioral variance, including APOE × sex, APOE × diet × HN, and diet × HN × sex × age (all *p* < 0.01). These findings support that diminished reward-seeking under olfactory stimulation is shaped by genotype-immune interactions, and modulated by age, sex, and diet.

Anhedonia-like behavior, defined as the proportion of total tracked time that mice spent not engaging with odorized objects, showed distinct genotype-specific age effects. Estimated marginal means (averaged over diet, sex, and HN status) indicated that APOE2 mice increased slightly from 0.79 [95% CI: 0.75–0.82] at 12 months to 0.81 [0.78–0.84] at 18 months (+0.02, *p* = 0.34), and APOE3 mice decreased slightly from 0.84 [0.80–0.87] to 0.82 [0.78–0.85] (−0.02, *p* = 0.39); neither change was statistically significant. In contrast, APOE4 mice exhibited a significant age-related increase, from 0.76 [0.73–0.79] at 12 months to 0.82 [0.78–0.86] at 18 months (+0.06, *p* = 0.026). These results demonstrate that aging is associated with a significant increase in anhedonia-like behavior in APOE4 mice, whereas APOE2 and APOE3 mice show only nonsignificant shifts.

#### 3.1.3. Exploratory Dynamics

Cumulative exploratory behavior quantified via area under the curve (AUC) metrics demonstrated robust effects of age and environment (Figure S1). For normalized AUC, age had the strongest main effect (F(1, 1669) = 56.02, *p* < 0.001, η^2^_partial = 0.030), followed by diet (F(1, 1669) = 40.38, *p* < 0.001, η^2^_partial = 0.022) and sex (F(1, 1669) = 11.60, *p* < 0.001, η^2^_partial = 0.0064). Diet × age interaction (F(1, 1669) = 16.04, *p* < 0.001) and higher-order interactions (e.g., APOE × diet × HN × sex × age, η^2^_partial = 0.0097; APOE × HN × sex × age, η^2^_partial = 0.0095) reflected the complexity of modulatory influences. These findings suggest that signal integration across concentrations (as reflected in AUC) is highly sensitive to the interaction of genotype, immune status, and age. Raw AUC analysis confirmed these patterns, with APOE × HN again emerging as the strongest interaction (F(2, 1669) = 66.32, *p* < 0.001, η^2^_partial = 0.068). Main effects of age (F(1, 1669) = 77.99, *p* < 0.001), diet (F(1, 1669) = 45.58, *p* < 0.001), and sex (F(1, 1669) = 19.90, *p* < 0.001) were robust, alongside immune-related interactions (HN × sex and diet × HN, both *p* < 0.001). Figure S1 shows cumulative odor exploration, expressed as raw area-under-the-curve (AUC_raw). Across all three APOE genotypes, AUC_raw was significantly higher in 12-month mice compared to 18-month mice (F(1,1812) = 56.0, *p* < 0.0001, partial η^2^ = 0.03, Cohen’s f = 0.18), indicating a medium effect of age. Diet also exerted a notable influence (F(1,1812) = 40.4, *p* < 0.0001, partial η^2^ = 0.022, f = 0.15), with high-fat diet animals showing reduced odor exploration overall. The largest diet-related divergence was seen in 18-month APOE4 mice, in whom AUC_raw was markedly lower on the high-fat diet compared to age-matched controls, while diet effects were modest in APOE2 and APOE3 mice. APOE genotype contributed a smaller, yet significant effect (F(2,1812) = 7.1, *p* = 0.0008, partial η^2^ = 0.008, f = 0.09). Raw AUC thus provides a complementary metric of behavioral sensitivity to aging and immune factors, highlighting how environmental and genetic variables shape odor-guided behavior. These results suggest that the influence of APOE genotype on odor-preference and odor guided behavior is significantly modulated by both diet and immunity particularly in the context of aging. These results suggest compounded vulnerability in aging APOE4 mice on a high-fat diet.

#### 3.1.4. Habituation/Dishabituation

We analyzed exploration time across all four objects during repeated odor presentations (Trials 1–4) and upon odor substitution (Trial 5) to assess general habituation (Figure S2). Animals showed significant reduction in head time across trials (F(3,1240) = 106.94, *p* < 1.4 × 10^−61^, η^2^ = 0.21), consistent with robust habituation (Figure S2A). Diet (F(1,425) = 15.26, *p* < 0.0001, η^2^ = 0.035) and age (F(1,425) = 8.14, *p* < 0.005, η^2^ = 0.019) contributed independently to exploratory behavior, with older and HFD mice generally showing reduced engagement.

To isolate responses to the odor-switched object (Obj1), we examined head time directed specifically at Obj1 across trials (Figure S2B). A similar pattern of habituation was observed (F(3,1241) = 87.70, *p* < 1.8 × 10^−51^, η^2^ = 0.175), though with additional significant effects of APOE genotype (F(2,425) = 5.39, *p* < 0.005, η^2^ = 0.025), revealing genotype-specific modulation of odor investigation.

Dishabituation was evaluated by comparing Trial 5 head time directed at Obj1 (odor-switched) versus prior trials. While no main effects of genotype, age, or diet reached significance across all four objects or for Obj1 alone, significant three-way interactions emerged. Specifically, APOE × Age × Sex and APOE × HN × Sex interactions reached significance (*p* = 0.02 and *p* = 0.04, respectively; Figure S2C), with modest effect sizes (η^2^ ≈ 0.02).

Figure S3 shows the Dishabituation Index, where higher values indicate a stronger increase in sniffing toward the novel odor (No) relative to the final familiar exposure (Fo). In the 12 month cohort, DI values were similar across APOE2, APOE3, and APOE4, and HFD produced a modest overall increase relative to control diet. The 18-month-old cohort displayed a genotype-specific pattern: HFD continued to elevate DI in APOE2 and APOE3 mice, but the effect reversed in APOE4, where aged HFD animals showed a markedly lower DI than their control-fed counterparts, indicating a dishabituation response unique to the APOE4 + old age + HFD combination.

#### 3.1.5. Odor Recognition Memory Across Delay Intervals

**Figure 3:**
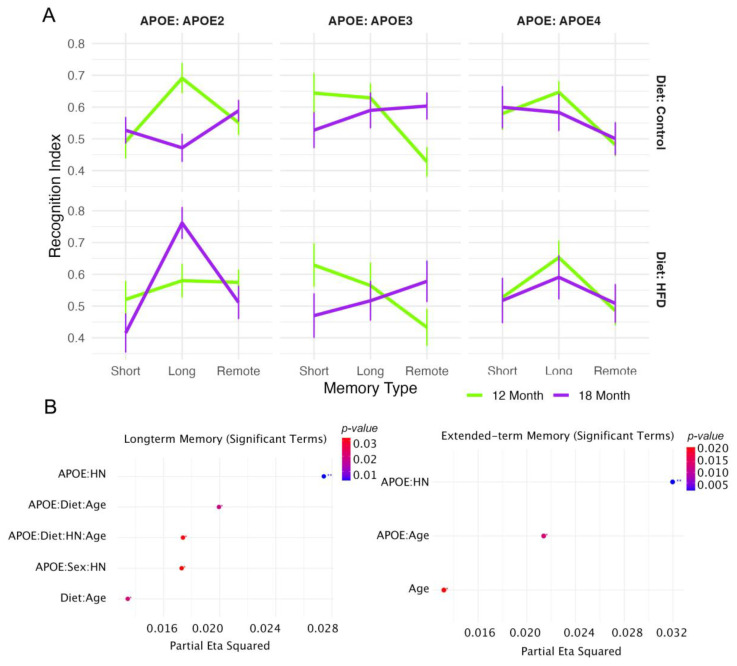
Odor recognition memory performance varies by APOE genotype, age, and diet across delay intervals. (**A**) Recognition Index (RI) was assessed after short-term (1 h), long-term (24 h), and extended-term (48 h) delays. Across genotypes, animals showed intact short-term memory (RI > 0.5). Long-term memory showed age- and genotype-specific effects, with APOE2 mice on a control diet displaying elevated RI at 12 months and reduced RI at 18 months. APOE3 and APOE4 groups showed flatter trajectories with diet- and age-associated decline. extended-term memory was impaired in all groups, especially with advancing age. (**B**) Forest plots show significant interaction effects on long-term and extended-term memory. Long-term recognition was significantly modulated by APOE × HN, APOE × Diet × Age, and Sex × HN interactions (partial η^2^ up to 0.028). extended-term memory showed significant contributions from APOE × Age and APOE × HN (partial η^2^ up to 0.032). Terms are sorted by η^2^, with significance stars indicating thresholds: * *p* <  0.05, ** p < 0.01.

Recognition index (RI) scores were used to evaluate short-term (1 h), long-term (24 h), and extended-term (48 h) odor memory across APOE genotypes, diets, and age groups (Figure 3A). While all groups displayed intact short-term memory (RI > 0.5), genotype and age-related differences emerged with longer delays. At 24 h, APOE2 control-fed mice at 12 months showed the highest RI, with age-associated decline at 18 months. APOE3 and APOE4 groups exhibited flatter or diminished performance across age and diet conditions.

Linear Mixed effects modeling revealed significant interactions for APOE × HN, APOE × Diet × Age × Category, and Sex × HN (Figure 3B; partial η^2^ = 0.028, *p* < 0.01), indicating sensitivity of long-term memory to both genetic and environmental modifiers. At 48 h, all groups showed reduced recognition, with APOE × HN and APOE × Age interactions reaching statistical significance (partial η^2^ = 0.032, *p* < 0.01), suggesting progressive deficits in odor memory consolidation and retrieval under risk-related conditions.

For short-term memory, the linear model revealed no significant trait-specific differences in RI. In the long-term memory test, several significant interaction effects were observed, including Age × Diet [F(1, 395) = 5.4, *p* = 0.02], APOE × HN genotype [F(2, 395) = 5.6, *p* = 0.004], Age × APOE × Diet [F(2, 395) = 4.0, *p* = 0.02], and APOE × Sex × HN [F(2, 395) = 3.5, *p* = 0.03]. For extended-term memory, Age was a significant factor [F(1, 398) = 5.3, *p* = 0.02], along with significant interactions between Age × APOE [F(2, 398) = 4.3, *p* = 0.01] and APOE × HN [F(2, 398) = 6.6, *p* = 0.002]. In the linear mixed-effects model that included memory type as a stage factor, both Age [F(2, 1179) = 4.9, *p* = 0.008] and APOE [F(4, 1179) = 2.8, *p* = 0.03] were significant main effects. Additionally, significant interactions were observed for Age × APOE [F(4, 1179) = 3.4, *p* = 0.009] and APOE × HN [F(4, 1179) = 5.7, *p* = 0.0001]. Overall, Age and APOE and their interaction emerged as consistent and significant factors across memory stages.

Genotype-specific trends revealed that APOE2 mice had lower RI in short-term memory but better retention across longer intervals, with the smallest drop from long-term to extended-term memory. APOE3 mice exhibited a pronounced age-related decline in extended-term, while APOE4 mice showed the least improvement from short- to long-term memory and the greatest loss from long-term to extended-term memory at 18 months, indicating greater vulnerability with age compared to APOE2 and APOE3.

### 3.2. Multimodal Clustering Reveals Risk-Linked Olfactory–Limbic Networks

**Figure 4:**
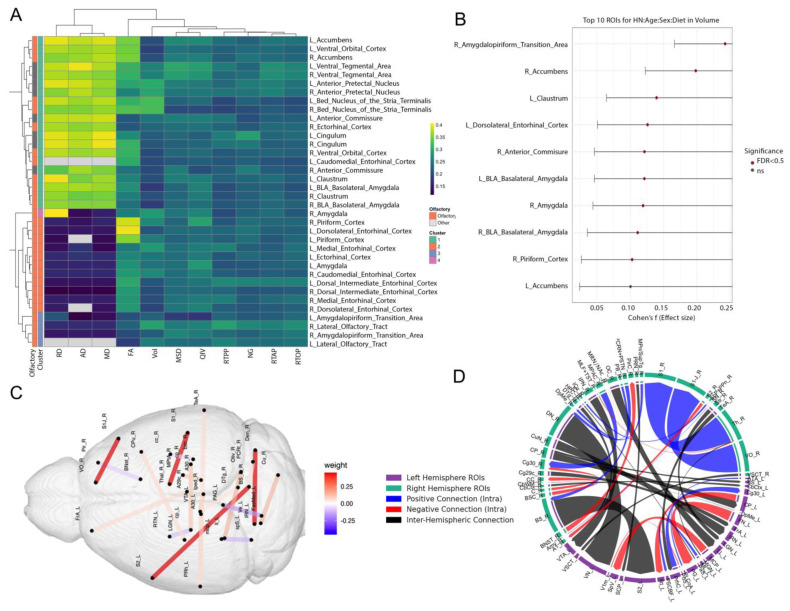
Clustering of olfactory-memory ROIs and top region effect sizes. (**A**) Hierarchical clustering of 18 a priori–selected olfactory–memory regions based on their Cohen’s f effect sizes across 11 imaging metrics. Branch lengths reflect Euclidean distance. Each row represents a brain ROI, and each column corresponds to a DTI metric: fractional anisotropy (FA), radial diffusivity (RD), axial diffusivity (AD), mean diffusivity (MD), volume percentage (Vol), or MAP metric (MSD, QIV, RTPP, RTAP, RTOP, NG). ROIs are annotated by cluster membership (left bar) and olfactory categorization (second left bar), with olfactory ROIs marked in orange and others in light gray. Note that cluster 2 was highly enriched for core olfactory areas, e.g., iriform cortex, amygdalopiriform transition area, but also entorhinal cortices, indicating these regions share a distinct signature of AD-risk effects. Cluster 4 captured more associative/hub regions (ventral orbital cortex, nucleus accumbens). (**B**) Forest-style dot plot of the top ten regions ranked by absolute Cohen’s f (meta-analysis across risk factors), showing point estimates for regional relative volumes (colored by olfactory vs. other) and 95% confidence intervals. The amygdalo-piriform transition area and nucleus accumbens exhibited the largest effect sizes (f ≈ 0.24 and 0.20, respectively), underscoring their vulnerability to combined APOE, diet, age, sex, and immune-background influences. Several canonical olfactory structures (piriform cortex, basolateral amygdala, anterior commissure) also ranked among the top ten, highlighting that alterations in primary olfactory nodes parallel those in downstream reward and associative circuits. (**C**) Top 20 Elastic MCCA connectivity weights, included connections for the Ventral Orbital Cortex (VO), Primary Somatosensory Cortex Jaw Region (S1J), Brain Stem rest (BS), Secondary Somatosensory Cortex (S2), Dentate Nucleus of Cerebellum (DN), Vestibular Nuclei (Ve), Thalamus (Thal), Primary Somatosensory Cortex (S1), Fastigial Medial Nucleus of Cerebellum (FastMed), Periaqueductal Gray (PAG), Olivary Complex (Olv), Lateral Lemniscus (LL), Cuneate Nucleus (Cu), Intermediate Reticular Nucleus (IRN), Interpeduncular Nucleus (IPed), Ventral Tegmental Area (VTA), Middle Cerebellar Peduncle (mcp), Spinal Trigeminal Nerve (sp5), Cerebral Peduncle (cp), Reticular Nucleus of Thalamus (RTN), Medial Parietal Association Cortex (MPtA), Cingulate Cortex (Cg), Perirhinal Cortex (PRh), Bed Nucleus of the Stria Terminalis (BNst), Piriform Cortex (Pir), Frontal Association Cortex (FrA), Striatum (CPu), Lateral Geniculate Nucleus (LGN), Temporal Association Cortex (TeA), Parvicellular Reticular Nucleus and Principal Sensory Trigeminal Nucleus (PCRt/sp5), Brachium of Superior Colliculus (bsc), Corpus Callosum (cc), Dorsal Tegmentum (DTg). (**D**) Chord diagram showing the top connections and their weights following elastic net MCCA.

A region of interest (ROI) analysis centered on 18 preselected regions revealed how these map in relation to each other, and the impact of risk factors on image metrics including volume, diffusion and mean apparent propagator parameters (Figure 4). The regions encompassed the olfactory system and associated limbic/memory circuits, including: Piriform Cortex, Amygdalopiriform Transition Area, Lateral Olfactory Tract, Ectorhinal Cortex, Caudomedial Entorhinal Cortex, Dorsal Intermediate Entorhinal Cortex, Dorsolateral Entorhinal Cortex, Medial Entorhinal Cortex, Amygdala (including Basolateral Amygdala), Accumbens, Claustrum, Cingulum, Ventral Orbital Cortex, Anterior Commissure, Bed Nucleus of the Stria Terminalis, and Ventral Tegmental Area. A hierarchical clustering based on Euclidean distance and complete linkage revealed that several clusters are enriched in olfactory regions, indicating shared effect size patterns across metrics in these structures. These analyses show that (1) primary olfactory regions cluster together based on shared patterns of imaging changes under AD risk, and (2) the strongest risk-associated effects occur not only in canonical olfactory regions but also in limbic and reward-related regions that integrate odor information with memory and motivational valence.

Elastic MCCA identified brain networks associated with risk factor traits including age, APOE genotype, diet, sex, and HN genotype as well as odor-based behavioral metrics. The sum of correlations was 1.267, with 1000 bootstrap iterations yielding a 95% confidence interval of [1.183, 1.846] and a *p*-value < 0.0001. Following Elastic Net regularization, 242 connections were retained, which were further reduced to 39 through MCCA. Applying a threshold to exclude connections with weights below 0.05 resulted in a final set of 20 connections (Figure 4, Table S3). Among these, the strongest weighted connections (weight = 0.452) included: right brainstem to left secondary somatosensory cortex, right dentate nucleus of the cerebellum to left vestibular nuclei, right thalamus to right primary somatosensory cortex, and right ventral orbital cortex to right primary somatosensory cortex. The next highest absolute weight was observed between the left fastigial medial nucleus of the cerebellum and the left periaqueductal gray (weight = −0.158). The presence of both positive and negative weights suggests a combination of strengthened and compensatory connections associated with increased scent preference and novel odor recognition index.

### 3.3. Transcriptomic Modules and Their Coupling with Brain Microstructure

#### 3.3.1. Functional Enrichment of Blood-Derived Gene Modules

To investigate whether peripheral markers of gene expression reflect changes in olfactory-memory circuits, we first performed transcriptomic analyses from whole blood RNA using PCA, which identified 10 modules which we tested for co-expressed gene clusters. Functional enrichment analysis revealed several modules that were biologically meaningful, particularly in the context of olfaction and memory. As shown in Table 2, Eigengenes 1 to 3 showed the most prominent enrichment for biologically relevant pathways. Eigengene 1 was significantly associated with pattern recognition receptor signaling (*p* = 0.003), suggesting involvement in early sensory detection and immune-like receptor activity. Eigengene 2 was enriched for phagocytosis and recognition-related pathways (*p* = 0.007), implicating roles in sensory clearance and responsiveness to external stimuli. Notably, Eigengene 3 showed strong enrichment for learning and memory functions (*p* = 0.002), including processes involved in hippocampal development—highlighting a direct link between gene regulation and cognitive mechanisms underlying olfactory memory formation. Eigengenes 4–8 demonstrated moderate enrichment for pathways such as neuron recognition, cell–cell signaling, and receptor activity. Eigengene 4 was associated with neuron recognition (*p* = 0.010), while Eigengene 8 showed enrichment for cell adhesion involved in neuronal migration (*p* = 0.029), suggesting supportive roles in neuronal connectivity and communication.

#### 3.3.2. Gene Modules Predict Regional Microstructure in Olfactory–Memory Circuits

To assess whether specific gene expression modules covary with local brain structure, we examined correlations between blood-derived eigengenes and regional microstructural MRI metrics in the same animals, across eight predefined olfactory-memory regions. We identified 431 significant eigengene–region correlations (FDR < 0.05), highlighting widespread and spatially specific molecular–structural coupling within these circuits. The strongest correlations were observed in the Parasubiculum, where diffusion-based metrics—including axial diffusivity (AD), radial diffusivity (RD), and mean diffusivity (MD)—were tightly linked to Eigengene 2 (Table 3). The top five absolute correlations (|r| ≈ 0.5) all involved Eigengene 2 and were highly significant (FDR-adjusted *p* < 1 × 10^−30^), with R^2^ values indicating that 24% of the variance in regional diffusivity could be explained by this eigengene.

Similar associations were found in the Hippocampus and Postsubiculum and Piriform Cortex, underscoring the relevance of Eigengene 2 to microstructural variation across the subicular–hippocampal axis. Across the Parasubiculum, Hippocampus, and Postsubiculum, Eigengene 2 expression was strongly and inversely related to diffusion indices. AD, RD, and MD all showed correlations of r ≈ −0.48 to −0.49, while FA was positively associated, and every relationship remained highly significant after correction (FDR < 1 × 10^−31^). These results indicate that higher Eigengene 2 expression co-occurs with lower diffusivity and thus better tissue integrity, and thus neuroprotection in key memory-related regions. Eigengene 2 emerged as a driver of structural variability within deep olfactory-memory regions, particularly those implicated in memory encoding and contextual sensory integration. Other eigengene modules demonstrated more regionally restricted associations, e.g., Eigengene 3 showed moderate correlations (r ≈ 0.3–0.4) with diffusion metrics in the Piriform Cortex, while Eigengene 4 was preferentially associated with the Ventral Orbital Cortex. These results suggest modular specificity in how transcriptomic variation relates to local brain architecture across the olfactory-memory system. Interestingly, volume-based metrics yielded weaker associations overall (|r| < 0.3), suggesting that diffusion MRI is more sensitive than regional volumetry in capturing molecular influences on tissue structure. This supports the utility of advanced diffusion metrics in detecting subtle gene-related variability in brain architecture.

Eigengene 2 captured molecular processes, potentially related to lipid metabolism, myelination, or neuroinflammatory signaling, tightly coupled to microstructural integrity in key olfactory-memory hubs such as the subiculum and hippocampus. Modules such as Eigengenes 3, 4, 5, and 8 appeared to mediate structural variation in more anterior olfactory regions, including the Piriform and Ventral Orbital Cortices. The breadth of significant eigengene–MRI associations reflect both the biological richness of the blood transcriptome and the sensitivity of diffusion imaging to detect microstructural changes relevant to olfactory behavior and memory.

Across modules, the top loading genes highlighted a rich interplay between synaptic plasticity (Grin2a, Camk2a, Snap25), metabolic support (Cs, Cox5a, Uqcrc2), glial modulation (Gfap, S100b), and myelination (Plp1, Mog). Multiple genes (e.g., Nptx2, Camk2a, Grin2a) have links to both memory and olfactory processing, suggesting these eigengene modules capture biologically relevant axes of neural function underlying sensory memory and circuit plasticity.

#### 3.3.3. Brain Regions–Blood Transcriptomic Coupling

**Figure 5:**
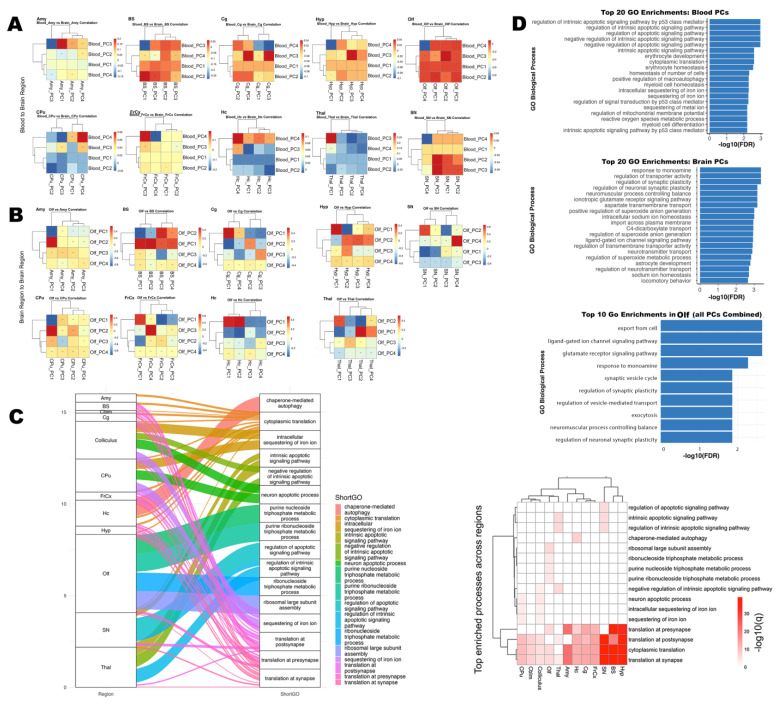
Mapping Peripheral to Central Transcriptomic Processes. (**A**) Heatmaps illustrating the correlation between blood and brain PCs for amygdala (Amy), brainstem rest (BS), cingulate cortex (Cg), hypothalamus (Hyp), Olfactory areas/bulbs (Olf), caudate putamen (CPu), frontal cortex (FrCx), hippocampus (Hc), thalamus (Thal), substantia nigra (SN). * *p* < 0.05. (**B**) Heatmaps illustrating the correlation between PCs from olfactory and other brain regions. * *p* < 0.05; ** *p* < 0.01. (**C**) Sankey diagram depicting pathways derived from genes driving enriched pathways for significantly correlated PCs between brain regions and blood (top), and clustered heatmap showing convergence of blood to region-specificity of enriched biological processes. (**D**) Barplots showing the top enriched biological processes for blood PCs associated with brain regions, brain region–to–brain region; and olfactory region to blood coupling analyses. Bars represent –log10(FDR) for each process.

To elucidate the molecular coupling between the brain and peripheral blood, and then between olfactory and other brain regions, we performed PCA on transcriptomes from each compartment (Figure 5A,B). For each brain region, the top four PCs were computed, and we assessed pairwise correlations between Olf PCs and those from other regions or blood samples. Our analyses identified numerous significant correlations (FDR < 0.05) between specific Olf PCs and PCs from other brain regions (Figure 5B), as well as between Olf PCs in blood and PCs from other regional blood samples (Figure 5A). The strongest associations were found for olfactory–hippocampus and olfactory–thalamus PC pairs, with both positive and negative correlations observed across principal axes. These patterns indicate that only a subset of major transcriptomic axes underlie inter-regional and brain–blood coupling, reflecting targeted, rather than global, coordination of gene expression programs.

#### 3.3.4. High-Loading Genes Reveal Shared Molecular Drivers

We identified genes that contributed most to the observed correlations for the significant PC pairs, based on absolute PC loadings. GO enrichment analysis of these high-loading genes revealed convergence on pathways involved in apoptotic signaling, erythrocyte development, oxidative stress response, synaptic plasticity, and transporter activity.

In blood, several canonical regulators of oxidative defense and metabolism—including Gpx1, Sod2, Nqo1, and Slc2a1—were among the most highly loaded genes in significant PCs, underscoring the central role of redox and metabolic homeostasis in mediating peripheral transcriptomic signals associated with brain states. Notably, blood PCs were also enriched for pathways related to synaptic plasticity, neurotransmitter signaling, and immune function, suggesting that blood transcriptomic profiles reflect key aspects of central nervous system activity and vulnerability.

Within the brain, significant PC pairs were driven by high-impact genes involved in apoptosis (Trp53, Casp3, Bax), synaptic regulation (Grin2b, Dlg4, Syn1), and metabolic stress (Sod1, Cat, Gpx1, Nrf2, Hmox1). The most significantly enriched biological processes included intrinsic apoptotic signaling (p53-mediated), cytoplasmic translation, synaptic transmission, and erythrocyte homeostasis. Several genes—including Gpx1, Nqo1, and Slc2a1—appeared in both blood and brain analyses, highlighting systemic coordination and potential shared molecular mechanisms.

Pathway coupling (Figure 5C) demonstrated that processes such as apoptosis, protein translation, synaptic function, and metabolic regulation were represented among the top genes across multiple brain regions. These pathways were enriched in several, but not all, region pairs, reflecting both global and region-specific molecular drivers.

Across the most significant blood–olfactory region PCs pairs, we observed strong enrichment for pathways involved in protein synthesis, metabolic regulation, and cellular transport. The leading processes included cytoplasmic translation and translation at the presynapse, ribosomal large subunit biogenesis, carbohydrate derivative catabolic process, purine-containing compound metabolic process, and protein localization to cell–cell junctions. These findings highlight the shared regulation of translation and synaptic protein synthesis, as well as core metabolic pathways, between blood and olfactory brain tissue. The presence of these intersecting pathways suggests robust molecular links between peripheral and central compartments, including processes essential to olfactory neuronal function and supporting the idea that blood-based gene expression can reflect brain-region vulnerabilities.

We visualized the top 20 significantly enriched GO biological processes for brain and blood coupling and brain region to region coupling (Figure 5D). In blood, intrinsic apoptotic signaling, cytoplasmic translation, erythrocyte development and homeostasis, and synaptic plasticity were among the most enriched pathways. In brain, enrichment included apoptotic processes, canonical neural pathways (regulation of synaptic plasticity, ionotropic glutamate receptor signaling, monoamine response), transporter activity, immune processes, and oxidative stress responses. Several pathway, e.g., oxidative stress response, iron ion sequestration, and metabolic homeostasis were common to both compartments, underscoring their centrality in coordinated molecular responses.

Collectively, these results demonstrate that transcriptomic coupling between olfactory, other brain regions, and blood is driven by a core set of high-impact genes acting within key biological pathways, including apoptosis, synaptic regulation, metabolic, and oxidative stress responses. The broader view provided by the top 20 enrichments further underscores the diversity and complexity of the processes mediating brain–blood communication and regional brain coordination. The convergence of these processes across compartments supports a model of integrated molecular signaling underpinning both regional brain interactions and brain–blood communication.

#### 3.3.5. Synaptic and Ion Regulation Pathways Shared by Blood and Olfactory Brain Regions

To further dissect the molecular mechanisms linking brain and blood, we prioritized pathways and genes relevant to the olfactory region (Figure 5D lower panel), focusing on biological processes that exhibited significant shared variation between olfactory brain tissue and blood gene expression. GO enrichment analysis of the top intersecting genes revealed robust enrichment for pathways essential to olfactory function, most notably the glutamate receptor signaling pathway and ligand-gated ion channel signaling pathway (FDR-adjusted *p* = 0.0023). These pathways are fundamental for synaptic transmission within olfactory circuits, where glutamatergic signaling is central to conveying odor information from olfactory sensory neurons to the olfactory bulb and higher-order brain regions. In addition to glutamatergic signaling, we found significant enrichment for biological processes such as regulation of synaptic plasticity, intracellular sodium and potassium ion homeostasis, and regulation of neurotransmitter transport. These mechanisms are critical for olfactory signal transduction and support the adaptive capacity of olfactory networks during odor learning and memory. The enrichment of these terms suggests that shared molecular signatures between brain and blood are rooted in processes fundamental to olfactory system function. Several of the top shared genes are implicated in olfactory neuronal function and synaptic physiology. For example, Atp1a3 maintains neuronal ion gradients necessary for action potential propagation within olfactory pathways [42], while App is involved in synaptic organization and function [43] and is expressed in olfactory regions. Plp1 supports myelination and rapid signal conduction in the olfactory system [44], and Cplx2 regulates neurotransmitter release at olfactory synapses [45]. These results highlight a molecular bridge between peripheral blood and olfactory brain regions implicating synaptic signaling, ion homeostasis, and neurotransmitter regulation as shared processes across tissues. Our findings reinforce the role of glutamatergic and synaptic pathways in olfactory function, and demonstrate that blood-based molecular signatures can reflect region-specific vulnerabilities.

## 4. Discussion

We found that AD risk factors (age, APOE and HN genotypes, diet, and sex) exert distinct, often interactive, effects on odor-guided and memory-related behaviors and associated bran circuits, highlighting the complexity of sensory–cognitive processes. One of our most striking findings was that different odorant (lemon) concentrations elicited distinct patterns across risk factors. Older mice generally showed higher preference for lower odor concentrations, while younger mice were more responsive to higher concentrations. These patterns may reflect age-related hyposmia, which elevates detection thresholds. Stronger odors, when detected, can also become aversive, reducing investigation time [46]. Motivational or exploratory differences could also contribute, with older mice engaging more with weaker stimuli, and younger mice exhibiting greater novelty-seeking behavior at stronger concentrations [47]. Prior studies have shown that mice prefer lemon over other scents, supporting its salience as a behaviorally relevant odor [48].

Our study offered insight into how different APOE genotypes adapt to repeated stimuli and respond to novelty. During the habituation phase, APOE2 mice exhibited the lowest investigation time, suggesting more rapid adaptation. In the dishabituation trial, APOE2 mice showed higher dishabituation compared to APOE3 and APOE4 mice, under both control and high-fat diet (HFD). The difference between APOE2 and other genotypes, especially APOE4, became more pronounced under HFD conditions. This suggests that while APOE4 mice may be more sensitive to the detrimental effects of HFD, APOE2 mice could exhibit compensatory or protective responses [49,50]. These findings support the concept of genotype-specific dietary vulnerabilities or resilience, reinforcing the potential for targeted dietary interventions to mitigate or delay sensory–cognitive decline in genetically at-risk populations [51]. Our results suggest that APOE2 mice habituate more efficiently and sustain a strong novelty response—traits that may reflect neuroprotective mechanisms. Our results align with research indicating that APOE2 confers resilience against neurodegenerative processes [50,52]. In contrast, APOE4 mice exhibited slower decline in object-investigation time across trials, and blunted novelty responses, potentially reflecting reduced synaptic plasticity and impaired sensory processing associated with their genetic vulnerability [53,54].

Short-, long-, and extended-term memory performance provided further evidence of how genotype and age differentially influence memory consolidation and retrieval. Younger mice generally outperformed older mice in short- and long-term memory tasks but showed reduced performance in extended-term memory tests. While it is typical for younger animals to excel during initial encoding and consolidation phases, the divergence in extended-term memory in older cohorts may reflect trade-offs or compensatory mechanisms [55,56,57]. Interestingly, APOE2 mice exhibited lower short-term memory performance but higher extended-term memory retention with aging compared to other genotypes. This suggests that memory formation, consolidation, and retrieval may rely on partially distinct neural circuits whose efficiency varies by genotype and with age [50]. Our findings highlight the need for further investigation using electrophysiological or imaging methods to better understand how hippocampal-to-cortical transitions during extended-term memory processing are shaped by APOE status and aging.

Building on the behavioral results, elastic MCCA revealed that age, APOE genotype, diet, and sex jointly shape a set of brain networks spanning olfactory, limbic, and reward-related regions. These networks explained 24% variance in odor-guided behaviors, linking specific connections—e.g., involving the ventral orbital cortex and piriform cortex—to differences in novelty detection and memory. Connections with positive MCCA weights may support odor recognition and associative learning; negative weights may reflect compensatory or inhibitory pathways reorganizing in response to stressors [58]. Notably, the connection between the right ventral orbital cortex and the right primary somatosensory cortex exhibited one of the highest weights (0.45). The ventral orbital cortex plays a role in assigning reward value to odors, integrating olfactory information with other sensory cues to guide behavior [59]. The primary somatosensory cortex, traditionally associated with processing touch, temperature, and proprioception, has also been implicated—albeit speculatively—in cross-modal integration [4,5].These regions may support the formation and retrieval of associations between olfactory stimuli, spatial context, and the motor components of exploratory behavior [60].

In addition to the ventral orbital cortex–primary somatosensory cortex pathway, other connections emerged that further highlight the interplay among olfactory, emotional, and reward circuits. We noted, the bed nucleus of the stria terminalis, an extended amygdala structure crucial for regulating stress and anxiety [61], and the piriform cortex, involved in odor discrimination and memory formation [62]. The negative correlation (−0.072) between these areas suggests that as piriform cortex function or connectivity declines, stress pathways regulated by the bed nucleus of the stria terminalis may become disinhibited, linking diminished olfactory processing to heightened emotional reactivity [63]. Another notable connection was between the fastigial medial nucleus of the cerebellum and the periaqueductal gray (−0.158). The fastigial medial nucleus helps coordinate motor and affective processes [64], whereas the periaqueductal gray is central to defensive and autonomic responses [65]. The negative correlation could indicate that as the cerebellar nucleus modulates sensorimotor and emotional integration, periaqueductal gray-mediated defensive reactivity is reduced, or vice versa, reflecting changes in autonomic and visceral responses to odors [66]. Lastly, the interpeduncular nucleus and the ventral tegmental area demonstrated a positive correlation (0.137). The interpeduncular nucleus, located in the midbrain, has been implicated in reward processing, mood regulation, and certain olfactory-driven behaviors [67,68], while the ventral tegmental area is a dopaminergic hub integral to motivation and reward [32]. This connection, confirmed by viral tracing, undergoes synchronized activation for encoding motivated exploration, potentially increasing motivational salience or attentional focus on novel or preferred scents. [69]. By pinpointing neural circuits linked to these behaviors—e.g., connections involving the ventral orbital cortex and primary somatosensory cortex—our study highlights potential targets for future interventions, such as genotype-informed dietary strategies and therapies aimed at sustaining critical brain networks. Together, our results show that age, APOE genotype, and diet shape olfactory preference, novelty detection, and memory processes. The fact that APOE2 animals remain relatively resilient under HFD aligns with literature suggesting its protective role against metabolic/neurodegenerative stressors [50,52].

There are several limitations, including differences in baseline exploratory drive, potential stress from handling, circadian factors, and variations in odor delivery, all of which could affect the metrics collected. Replication in independent cohorts would establish the robustness of these findings, and more direct measures of neural activity (e.g., fMRI or in vivo electrophysiology) during odor tasks could validate the connectivity patterns reported here [70,71]. Extending these results to human populations, especially those carrying different APOE alleles or at risk from dietary factors, may help identify early biomarkers and interventions for age-related cognitive decline [72]. By integrating odor-based behavioral measures and network-level connectivity, our multifaceted approach offers a deeper understanding of how risk factors and neural connectivity markers converge to shape odor-based cognitive decline.

Mapping blood transcriptomic eigengenes onto MCCA-defined networks revealed that the same regions influencing behavior were also strongly associated with peripheral gene modules enriched for synaptic plasticity, glutamatergic signaling, metabolic regulation, and myelination. For example, Eigengene 2 correlated with diffusion-based microstructural integrity in olfactory–memory circuits (e.g., hippocampus, piriform, parasubiculum), explaining up to 24% of local diffusivity variance. This molecular–structural coupling suggests that peripheral gene expression patterns may mirror, and perhaps modulate, the brain circuits governing olfactory behavior and memory.

The blood based eigengenes revealed modules enriched for synaptic transmission and plasticity, epigenetic and metabolic regulation, glial support, and myelination. Multiple modules highlighted genes essential for learning and memory (*Camk2a*, *Grin2a*, *Snap25*, *Ppp1r1b*) and for olfactory circuit function (*Apoe*, *Gfap*, *Snap25*), supporting their roles in integrating sensory and cognitive processes. PC 1 through 8 captured major axes of molecular variation associated with imaging properties of brain regions, each with distinct functional relevance to memory and olfactory circuits. PC1 was driven by genes related to chromatin structure and vesicle trafficking (e.g., *Nsd1*, *Wdr82*, *Vps35*), essential for neuronal gene regulation and synaptic remodeling underlying learning and memory [73]. PC2 featured core regulators of synaptic plasticity and signaling such as *Nptx2* and *Camk2a*, implicated in olfactory learning and spatial memory [74]. PC3 prioritized genes governing neurotransmission, especially synaptic vesicle machinery and NMDA receptor function (e.g., *Grin2a*, *Snap25*), fundamental for long-term potentiation and memory encoding [75,76]. PC4 highlighted axon guidance and reward pathway genes (*Dpysl2*, *Fyn*, *Ppp1r1b/DARPP-32*), with roles in dopaminergic signaling and memory circuits [77]. PC5 was enriched for ribosomal and elongation factor genes, supporting the protein synthesis required for memory consolidation and synaptic plasticity in both olfactory and hippocampal systems [78]. PC6 was dominated by mitochondrial electron transport genes (e.g., *Uqcrc2*, *Ndufa1*, *Cox5a*), reflecting metabolic needs during high-demand cognitive and sensory processing [79]. PC7 was defined by glial and lipid metabolism genes (*Gfap*, *Apoe*, *S100b*), highlighting the importance of astrocytic support and lipid regulation in both synaptic plasticity and olfactory function [80]. Finally, PC8 captured myelination and oligodendrocyte-associated transcripts (e.g., *Plp1*, *Mobp*), crucial for rapid axonal conduction and effective transmission of memory and sensory information [81]. Together, these modules illuminate the tightly coordinated molecular programs underlying memory and olfaction in the mammalian brain.

We then mapped blood transcriptomic eigengenes onto these MCCA-defined networks. This revealed that the same regions influencing behavior were also strongly associated with peripheral gene modules enriched for synaptic plasticity, glutamatergic signaling, metabolic regulation, and myelination. For example, Eigengene 2 correlated with diffusion-based microstructural integrity in olfactory–memory circuits (e.g., hippocampus, piriform, parasubiculum), explaining up to 24% of local diffusivity variance. This molecular–structural coupling suggests that peripheral gene expression patterns may mirror, and perhaps modulate, the brain circuits governing olfactory behavior and memory.

We identified significant associations between blood gene expression profiles related to actin cytoskeletal regulation and regional diffusion MRI metrics of brain microstructure. Pathways related to actin filament organization, actin polymerization, intracellular transport, and cytoskeletal remodeling were enriched across multiple eigengene modules. These findings suggest that peripheral regulation of cytoskeletal dynamics may reflect or influence microstructural brain properties detectable by diffusion imaging.

We leveraged a PC–based correlation framework to dissect the molecular relationships between the olfactory region, other brain regions, and peripheral blood, revealing that a small subset of PC are strongly correlated across brain regions, and between brain and blood, highlighting targeted, rather than global, coordination of transcriptomic programs. These correlations were both positive and negative, reflecting a sometimes-antagonistic regulation of gene expression across neuroanatomical boundaries. We found that a core group of high-impact genes drives both region-to-region and brain–blood correlations, supporting that key biological processes such as apoptosis, metabolic and oxidative stress response, and synaptic plasticity are central to neural system resilience and disease risk.

Gene ontology enrichment analysis demonstrated that the genes driving these correlated PC converge onto core biological processes, with remarkable consistency between brain and blood, with prominent shared pathways including intrinsic apoptotic signaling (notably p53-mediated), cytoplasmic translation, ribosomal assembly, and erythrocyte homeostasis. The repeated enrichment of erythrocyte-related processes suggests a neurovascular or systemic metabolic component to brain transcriptomic coordination, consistent with emerging evidence linking vascular and metabolic dysfunction to neurodegeneration. In the pool of shared brain region to brain regions pathways, we observed enrichment not only for apoptotic and metabolic pathways, but also for synaptic plasticity, glutamate receptor signaling, and monoamine response, supporting that peripheral blood transcriptomes capture echoes of central nervous system state.

These results align with and extend previous studies suggesting that blood transcriptomics can reflect aspects of brain pathology, particularly when leveraging dimension reduction approaches that capture coordinated variance [82]. The convergence of apoptotic, synaptic, and metabolic stress pathways across central and peripheral compartments underscores the complexity of neurodegenerative disease and highlights the utility of integrated analyses in identifying shared molecular mechanisms and potential biomarkers.

Top-loading genes within these significant PCs included mediators of apoptosis (Trp53, Bax, Casp3), synaptic function (Grin2b, Dlg4, Syn1), ribosomal and translational activity, metabolic/oxidative stress responses (Sod1, Cat, Gpx1, Nrf2, Hmox1). The high-loading genes associated with metabolic and oxidative stress—such as Gpx1, Sod2, Nqo1, and Slc2a1—underscore the contribution of redox and energy homeostasis to brain–blood transcriptomic coupling, and may represent critical nodes for cross-tissue signaling and biomarker development. The presence of neuron-centric pathways and genes in blood supports the potential of blood-based omics as accessible biomarkers for brain health and disease.

This study used a multistep analysis to connect behavioral, imaging, and molecular levels. First, Elastic MCCA identified brain networks—spanning olfactory, limbic, and reward-related regions—that explained variance in odor-guided behaviors across APOE genotype, age, diet, and sex. We then examined transcriptomic modules in many of these same regions—including the piriform cortex, hippocampus, and amygdala—revealing eigengenes linked to synaptic plasticity, ion channel signaling, and metabolic regulation. Blood-derived transcriptomic modules showed enrichment for synaptic plasticity, glutamatergic signaling, and oxidative stress, and covaried with microstructural integrity. The convergence between blood and brain signatures suggests that molecular signals detected in blood may provide biological context for the olfactory–limbic networks highlighted by MCCA, linking peripheral signatures to central circuit integrity. This stepwise framework—from behavior to brain networks to peripheral omics—helps contextualize and extend the imaging results, contributing to a unified systems-level perspective.

Limitations of this study include the potential for residual confounding due to technical variation or differences in cellular composition. Moreover, the PCA approach may underrepresent rare but functionally important cell-type–specific signals. Although the enrichment patterns we report plausibly underlie the age- and diet-dependent network changes, the RNA-seq cohort was not large enough to permit formal APOE × Diet × Age contrasts. An important next step will involve a targeted comparison of blood–brain transcript coupling in APOE4 mice exposed to both high-fat diet and advanced age, where our behavioral and imaging data show the greatest vulnerability. Larger, well-powered studies with expanded regional coverage and sequencing depth will be essential to confirm these relationships and clarify whether the shared glutamatergic, ion-homeostatic, and oxidative-stress genes identified here are further amplified or altered by genetic, metabolic, and aging effects. Nevertheless, the consistency of key pathways and genes across compartments, and across analytic approaches, strengthens the robustness of our findings.

Our integrative transcriptomic analysis identifies conserved and coordinated molecular programs underpinning brain–blood communication and regional brain interactions and captured synergistic as well as antagonistic relationships between regions and compartments. The central roles of apoptotic, metabolic, and synaptic pathways provide new insight into the mechanisms of neural resilience and vulnerability and highlight promising targets for biomarker discovery and intervention in neurodegenerative disease. By focusing on the top-loading genes from significantly correlated PC pairs between blood and brain, we have delineated biological processes that may drive coordinated changes between the olfactory system and broader neural circuits, as well as between central and peripheral compartments.

In summary, our findings show that age, APOE genotype, diet, and sex modulate odor-driven behaviors and memory in mice, with distinct concentrations revealing specific nuances in olfactory preference and exploratory drive. APOE2 mice appeared relatively resilient under high-fat diet conditions, whereas APOE4 carriers displayed patterns indicative higher vulnerability. Memory performance over short-term, long-term, and extended-term intervals underscored the interplay between genotype and age, suggesting differential effects on consolidation and retrieval. Elastic MCCA analyses identified key brain connections (notably involving the ventral orbital cortex and primary somatosensory cortex) that underpin odor recognition and associative memory, offering insight into how facilitative and compensatory circuits balance sensory and cognitive demands. Top correlated gene sets between olfactory region and brain were enriched for apoptosis and homeostasis, while in blood, correlated PCs unexpectedly revealed synaptic signaling and oxidative stress pathways—highlighting the potential for blood transcriptomics to reflect key neural processes relevant to AD risk. These observations point toward targeted interventions—such as genotype-appropriate dietary strategies or therapies—to preserve or enhance olfactory-based behaviors and cognitive health across the lifespan.

Our integrative analysis demonstrates that the molecular basis of olfactory–brain and brain–blood transcriptomic coupling is rooted in shared pathways of apoptosis, synaptic regulation, and metabolic homeostasis. The convergence of these processes across compartments and their association with high-loading genes reinforces their relevance to neural health and disease. These results provide mechanistic insight into brain–blood communication and highlight candidate targets for biomarker discovery and intervention in neurodegenerative disorders.

## 5. Conclusions

Our study demonstrates that olfactory-guided behaviors are sensitive to the combined effects of genetic and environmental risk factors for AD. Through integration of behavioral assays, neuroimaging, and transcriptomic profiling, we reveal that early alterations in sensory and cognitive processing are mirrored by changes in brain connectivity and peripheral gene expression. Our results highlight the potential of blood-derived transcriptomic modules—enriched for synaptic, metabolic, and immune-related pathways—to serve as accessible biomarkers reflecting central nervous system vulnerability. The convergence of molecular and systems-level signatures across olfactory and memory circuits underscores the value of integrative approaches in understanding the early mechanisms of neurodegenerative disease.

## Figures and Tables

**Table 1:** Behavioral Cohort Distribution by Genotype, Diet, Sex, Age Group, and NOS2 Background.

APOE	Diet	Female	Male	18 Months	12 Months	mNOS2	HN	Total
**APOE2**	Control	46	48	51	43	48	46	94
**APOE2**	HFD	37	34	31	40	29	42	71
**APOE3**	Control	45	41	41	45	43	43	86
**APOE3**	HFD	35	33	32	36	30	38	68
**APOE4**	Control	41	35	28	48	43	33	76
**APOE4**	HFD	39	31	23	47	34	36	70

**Table 2:** Top enriched biological pathways associated with eigengene modules for blood. Modules 1 to 3 show strong relevance to sensory recognition, 4 to 8 show neuronal signal and development.

Eigengene Module	Top Enriched Pathway	*p*-Value	Notes
Eigengene 1	Pattern recognition receptor signaling	0.003	Strong immune/sensory relevance
Eigengene 2	Phagocytosis, recognition	0.007	Sensory clearance processes
Eigengene 3	Learning or memory	0.002	Direct cognitive link (memory formation)
Eigengene 4	Neuron recognition	0.010	Supports neuronal differentiation
Eigengene 5	Pattern recognition receptor activity	0.015	Supports sensory input mechanisms
Eigengene 6	Cell surface receptor signaling	0.018	Moderate sensory/cognitive relevance
Eigengene 7	Cell–cell recognition	0.022	Intercellular signaling
Eigengene 8	Cell adhesion involved in neuron migration	0.029	Neuronal development process

**Table 3:** Top region-eigengene correlation between diffusion MRI metrics in olfactory-memory related structures and transcriptomic modules demonstrate a role for memory and olfaction related regions.

Structure	Metric	Eigengene	r	*p*_FDR	R^2^
Parasubiculum	AD	Eigengene 2	−0.4919	1.6 × 10^−32^	0.24
Parasubiculum	MD	Eigengene 2	−0.4870	8.6 × 10^−32^	0.24
Parasubiculum	RD	Eigengene 2	−0.4836	3.2 × 10^−31^	0.23
Hippocampus	AD	Eigengene 2	−0.4780	7.7 × 10^−31^	0.23
Postsubiculum	AD	Eigengene 2	−0.4777	8.1 × 10^−31^	0.23
Piriform Cortex	MSD	Eigengene 8	0.3636	2.1 × 10^−12^	0.14
Piriform Cortex	FA	Eigengene 2	0.3176	6.8 × 10^−13^	0.10
Piriform Cortex	NG	Eigengene 5	−0.3237	2.8 × 10^−11^	0.13
Piriform Cortex	RTOP	Eigengene 8	−0.3102	2.4 × 10^−9^	0.10
Piriform Cortex	RTOP	Eigengene 4	−0.3089	5.9 × 10^−11^	0.12
Hippocampus	MD	Eigengene 2	−0.4758	1.5 × 10^−30^	0.23
Hippocampus	RD	Eigengene 2	−0.4738	2.8 × 10^−30^	0.22
Hippocampus	FA	Eigengene 2	0.3689	2.7 × 10^−17^	0.14
Hippocampus	QIV	Eigengene 8	0.3643	2.1 × 10^−12^	0.14

## Data Availability

All data are available from the corresponding author upon reasonable request.

## Supplementary Materials

The following supporting information can be downloaded at: https://www.mdpi.com/article/10.3390/brainsci15080863/s1. Table S1: Imaging Cohort Distribution by Genotype, Diet, Sex, Age Group, and NOS2 Background; Table S2: Blood RNA Cohort Distribution by Genotype, Diet, Sex, Age Group, and NOS2 Backgrounds; Table S3: Elastic MCCA selected connectivity list and associated weights for the top graph edges. The connections are ranked from top to bottom based on absolute weight values; Figure S1: Overall odor exploration using AUC by APOE genotype, age category and diet; Figure S2. Effects of age, APOE genotype, and diet on olfactory habituation and dishabituation; Figure S3. Dishabituation time by APOE genotype, age category and diet served as an index of novelty detection.

## Abbreviations

The following abbreviations are used in this manuscript:

AD	Alzheimer’s disease
MCCA	Multi-set Canonical Correlation
APOE	Apolipoprotein E.
PC	Principal component
PCA	Principal component analysis
