# Supplementary material for: Olfactory-Guided Behavior Uncovers Imaging and Molecular Signatures of Alzheimer’s Disease Risk
Source: Brain Sci. 2025 Aug 13;15(8):863. doi: 10.3390/brainsci15080863 (PMC12384878; doi:10.3390/brainsci15080863)
Supplement: Supplementary file 1 [file brainsci-15-00863-s001.zip › Table_S1.pdf]

Table S1. Imaging Cohort Distribution by Genotype, Diet, Sex, Age Group, and NOS2 Background.

Genotype	Diet	Female	Male	18 Months	12 Months	mNOS2	HN	Total
APOE2	Control	23	21	34	10	34	10	44
APOE2	HFD	8	7	8	7	0	15	15
APOE3	Control	16	16	22	10	22	10	32
APOE3	HFD	14	12	11	15	13	13	26
APOE4	Control	20	22	28	14	27	15	42
APOE4	HFD	9	7	5	11	1	15	16
