# Supplementary material for: Olfactory-Guided Behavior Uncovers Imaging and Molecular Signatures of Alzheimer’s Disease Risk
Source: Brain Sci. 2025 Aug 13;15(8):863. doi: 10.3390/brainsci15080863 (PMC12384878; doi:10.3390/brainsci15080863)
Supplement: Supplementary file 1 [file brainsci-15-00863-s001.zip › Table_S2.pdf]

Table S2. Blood RNA Cohort Distribution by Genotype, Diet, Sex, Age Group, and NOS2 Background.

Genotype	Diet	Female	Male	18 Months	12 Months	mNOS2	HN	Total
APOE2	Control	22	19	28	13	34	7	41
APOE2	HFD	4	8	3	12	6	9	15
APOE3	Control	16	9	16	12	0	6	28
APOE3	HFD	11	8	6	13	0	6	19
APOE4	Control	7	16	15	10	0	8	25
APOE4	HFD	5	5	6	5	0	5	11
