# Supplementary material for: Olfactory-Guided Behavior Uncovers Imaging and Molecular Signatures of Alzheimer’s Disease Risk
Source: Brain Sci. 2025 Aug 13;15(8):863. doi: 10.3390/brainsci15080863 (PMC12384878; doi:10.3390/brainsci15080863)
Supplement: Supplementary file 1 [file brainsci-15-00863-s001.zip › Table_S3.pdf]

Table S3. Elastic MCCA selected connectivity list and associated weights for the top graph edges. The connections are ranked from top to bottom based on absolute weight values.

Region 1	Region 2	Weight
Right Ventral Orbital Cortex	Right Primary Somatosensory Cortex Jaw Region	0.452
Right Brain Stem	Left Secondary Somatosensory Cortex	0.452
Right Dentate Nucleus of Cerebellum	Left Vestibular Nuclei	0.452
Right Thalamus	Right Primary Somatosensory Cortex	0.452
Left Fastigial Medial Nucleus of Cerebellum	Left Periaqueductal Grey	–0.158
Right Olivary Complex	Left Lateral Lemniscus	0.144
Right Cuneate Nucleus	Left Intermediate Reticular Nucleus	0.143
Right Interpeduncular Nucleus	Left Ventral Tegmental Area	0.137
Left Middle Cerebellar Peduncle	Left Spinal Trigeminal Nerve	–0.117
Right Cerebral Peduncle	Left Cerebral Peduncle	0.115
Left Cerebral Peduncle	Left Reticular Nucleus of Thalamus	–0.103
Right Medial Parietal Association Cortex	Right Cingulate Cortex	0.101
Left Perirhinal Cortex	Left Cingulate Cortex	0.0837
Right Bed Nucleus of the Stria Terminalis	Right Piriform Cortex	–0.0717
Right Cingulate Cortex	Left Frontal Association Cortex	0.0684
Right Striatum	Left Lateral Geniculate Nucleus	0.0641
Right Temporal Association Cortex	Left Perirhinal Cortex	0.0630
Right Parvicellular Reticular Nucleus and Principal Sensory Trigeminal Nucleus	Right Brain Stem	0.0576
Right Brachium of Superior Colliculus	Right Corpus Callosum	–0.0528
Right Dorsal Tegmentum	Left Ventral Tegmental Area	0.0519
