# Supplementary figures and images for: Olfactory-Guided Behavior Uncovers Imaging and Molecular Signatures of Alzheimer’s Disease Risk
Source: Brain Sci. 2025 Aug 13;15(8):863. doi: 10.3390/brainsci15080863 (PMC12384878; doi:10.3390/brainsci15080863)

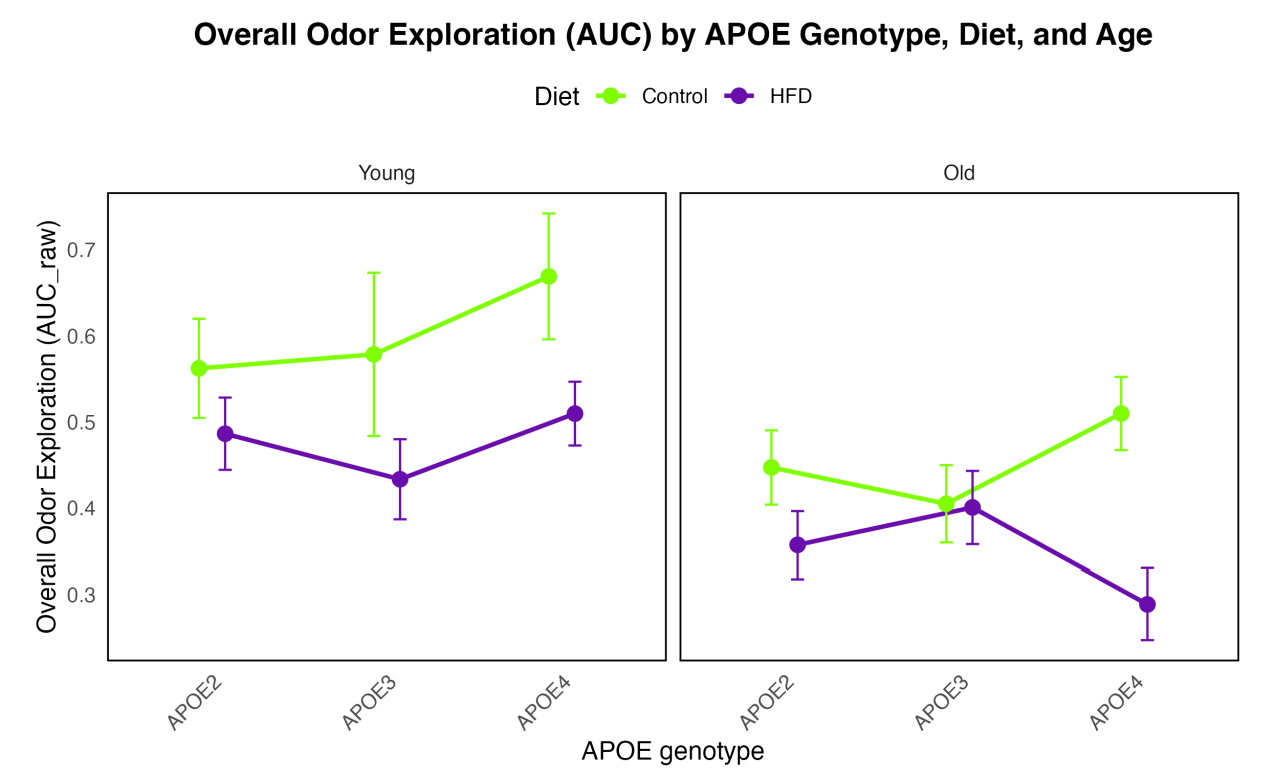

Supplement: Supplementary file 1 [file brainsci-15-00863-s001.zip › Figure_S1.png]

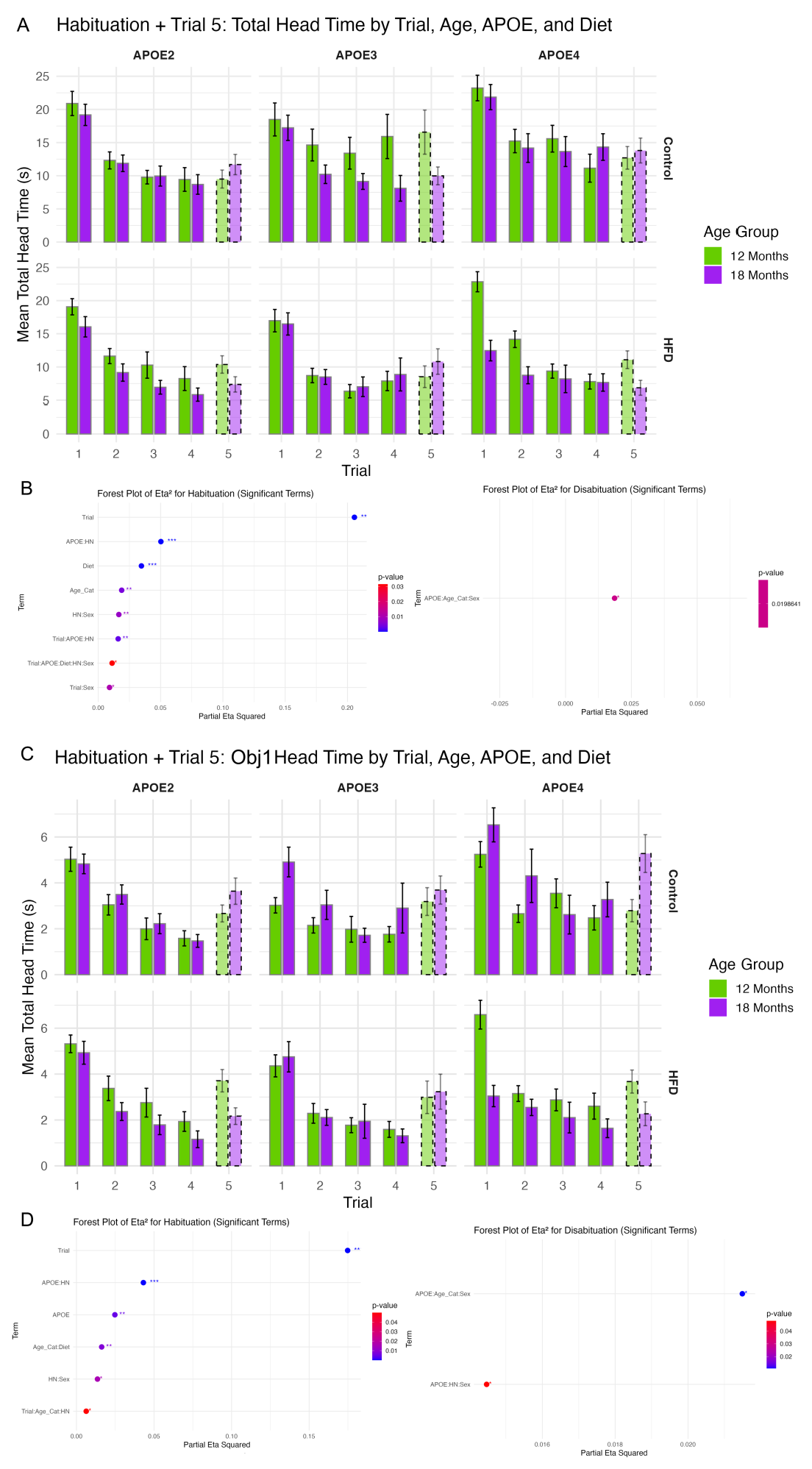

Supplement: Supplementary file 1 [file brainsci-15-00863-s001.zip › Figure_S2.png]

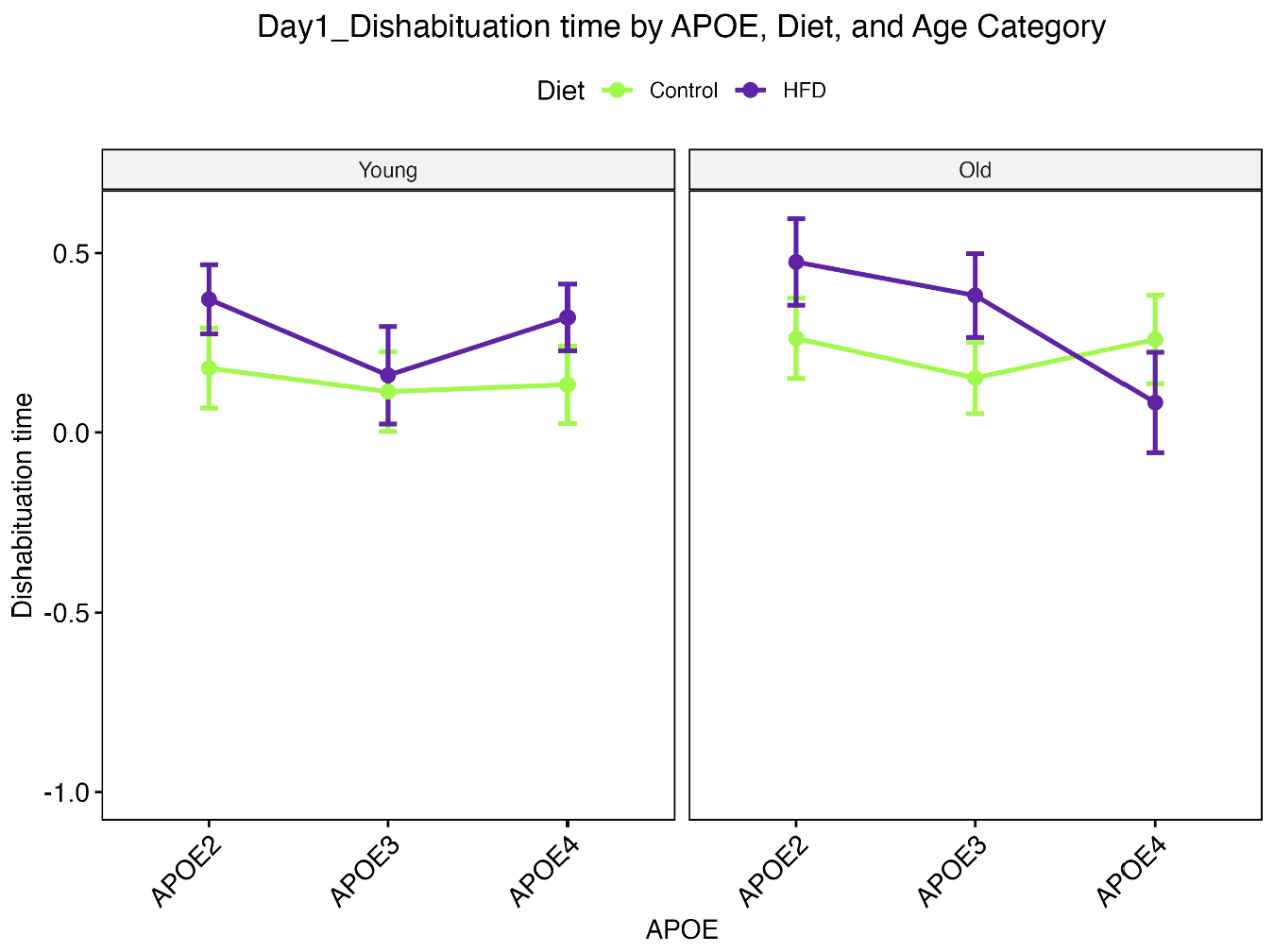

Supplement: Supplementary file 1 [file brainsci-15-00863-s001.zip › Figure_S3.png]
